# Dissecting the role of NETosis-related biomarkers in Sepsis: An integrated multi-dataset analysis for diagnostic and prognostic applications

**DOI:** 10.1371/journal.pone.0336244

**Published:** 2025-11-19

**Authors:** Binming Qiu, Xue Zhang, Huanlan Chen

**Affiliations:** 1 Department of Anesthesiology, Baiyun District People’s Hospital of Guangzhou, Guangzhou, Guangdong, China; 2 Department of Obstetrics, The Second Affiliated Hospital, Guangzhou Medical University, Guangzhou, Guangdong, China; Columbia University Irving Medical Center, UNITED STATES OF AMERICA

## Abstract

Sepsis is a life-threatening condition with high mortality and economic burdens. The study analyzed non-redundant differentially expressed genes (DEGs) to elucidate neutrophil extracellular trap (NET) formation’s role in sepsis pathogenesis using high-throughput microarray and bioinformatics. Our comprehensive analysis meticulously identified a total of 629 DEGs, encompassing 348 upregulated and 281 downregulated genes. Through further scrutiny, we discovered 37 NETosis-related differentially expressed genes (NRDEGs) that showcased distinct expression patterns. Enrichment analysis vividly revealed the significant involvement of these NRDEGs in pathways related to NET formation, phagocytosis, and lymphocyte migration, thereby highlighting the crucial role of neutrophils in the immune response during sepsis. Additionally, CIBERSORT algorithm analysis indicated substantial differences in the abundance of 17 immune cell types between the sepsis and control groups, further reinforcing the altered immune landscape in sepsis patients. A protein-protein interaction (PPI) network constructed from the NRDEGs identified nine core genes, suggesting their potential central position in the pathophysiology of sepsis. Receiver operating characteristic (ROC) curve analysis demonstrated that *ITGAM*, *CXCR*2, and *FCGR*3*B* exhibited extremely high accuracy in distinguishing sepsis from controls (with an area under the curve greater than 0.9). These remarkable findings strongly underscore the potential of these genes as biomarkers for early diagnosis and therapeutic targets in sepsis, emphasizing the urgent need for further validation in clinical settings to enhance diagnostic accuracy and refine treatment strategies. Overall, this study provides novel insights into the molecular mechanisms underlying sepsis, paving the way for improved clinical interventions.

## Introduction

Sepsis is a life-threatening organ dysfunction caused by a dysregulated host response to infection, resulting in fever, increased heart rate, difficulty in breathing, and other symptoms. It can also progress to septic shock and multiple organ failure, making it a significant cause of health morbidity and mortality worldwide [1]. In 2017, an estimated 48.9 million cases of sepsis were recorded worldwide and 11.0 million sepsis-related deaths were reported, representing 19.7% of all global deaths [2]. At present, the main treatment methods for sepsis include fluid resuscitation, appropriate antibiotics [3] and antiplatelet therapy [4], etc. Treatment limitations and the high socioeconomic burden of sepsis make it a major clinical challenge. However, the complex pathophysiology of sepsis is not fully understood. Therefore, it is essential to study the mechanism of its occurrence and delve into the key genes for clinical diagnosis and therapy.

Neutrophils represent the most abundant effector cells of the human immune system and have antibacterial and pro-inflammatory functions [5]. In the development of sepsis, neutrophils play a critical role in the host’s inflammatory response against invading pathogens [6,7]. Their exertion of effector functions is primarily achieved through three approaches: phagocytosis, degranulation, and the release of neutrophil extracellular traps(NET). NET neutralize and kill bacteria, viruses, and fungi, and could inhibit their dissemination [8]. However, if dysregulated, excessive NET could further induce inflammation and organ injury contributing to the progression of sepsis [9]. While there has been some attention given to the role of NET in sepsis, the crucial genes associated with NETosis in sepsis remain incompletely understood and are urgently required to be explored.

The aim of this study is to identify potential key genes related to NETosis in sepsis patients. As few prior research has explored specific gene and functional roles in regulating NETosis in sepsis patients, we utilized the Gene Expression Omnibus (GEO) database and integrated multiple bioinformatics approaches. Our focus is on discovering new findings in the molecular mechanisms of sepsis, which could be beneficial for discovering new therapeutic targets and providing effective strategies for the diagnosis and treatment of sepsis.

## Materials and methods

### Microarray data

We downloaded GSE134347 and GSE26440 microarray expression matrices (The datasets were accessed on June 29, 2024. All data were publicly available and anonymized, containing no identifiable participant information) from the GEO database (https://www.ncbi.nlm.nih.gov/geo/), The GEO database is an internationally recognized public repository that extensively archives high-throughput gene expression and genomic datasets from multiple species. It has become a widely utilized data source in biomedical research) and extracted the sepsis blood expression of Homo sapiens. The chip platforms of data sets GSE134347 and GSE26440 were GPL17586 (Illumina HumanHT-12 V4.0 expression beadchip) and GPL570 (Affymetrix Human Genome U133 Plus 2.0 Array), respectively, as shown in Table 1. Both platforms complied with MIAME standards, with peripheral blood samples processed through standardized workflows covering RNA extraction, specimen handling, microarray hybridization, and data normalization. Sepsis definitions followed original publications (Sepsis-3 criteria for GSE134347; AAP/SCCM consensus definition for GSE26440). The datasets GSE134347 and GSE26440 used in this study were sourced from the publicly available GEO database, with detailed sample information referenced to the original publications. The GSE134347 dataset included adult patients diagnosed with sepsis (predominantly bacterial infections, potentially viral or fungal) in the Intensive Care Unit (ICU), non-infectious critically ill patients (e.g., trauma, surgery), and healthy volunteer controls. The GSE26440 dataset comprised pediatric patients with septic shock (primarily bacterial infections) in the Pediatric Intensive Care Unit (PICU) and healthy child controls. Healthy controls in both cohorts were individuals without major diseases or inflammatory conditions, as confirmed by medical examinations. As the data originated from public databases and relied on the original studies [10,11], detailed clinical characteristics (e.g., disease severity scores, underlying comorbidities, immunosuppressive status) were not further characterized herein; such information is available in the respective source publications. This study focused on a comparative analysis of molecular expression profiles between sepsis patients and healthy volunteers. GSE134347 contained 156 sepsis data and 83 control data, and GSE26440 contained 98 sepsis data and 32 control data. All sepsis cases and controls were included in this study. These microarray data were collected and combined (Combined Dataset) using the "combat" method in the "sva" (Version 3.50.0) package in software R, which could remove batch effects. A total of 369 human peripheral blood samples were obtained, including 115 control data (Control) and 254 experimental data (Sepsis). Finally, Combined Dataset were standardized by R package limma (Version 3.58.1), and the annotation probes were calibrated, normalized, and transformed using log2. The expression of the matrix before and after the removal of the batch effect was subject to Principal Component Analysis (PCA) to verify the removal of the effect of the batch effect. Meanwhile, we downloaded neutrophilic inflammation cell death related genes (NETosis-related genes, NRGs) from the GeneCards database (https://www.GeneCards.org/). After searching the keyword "NETosis" in the GeneCards database, we retained 80 NRGs by setting "Protein Coding" and "Relevance Score>1". In addition, after searching "NETosis" as a keyword in the PubMed website (https://pubmed.ncbi.nlm.nih.gov/) in the published literature, a total of 69 NRGs were obtained. After merging and duplication removal, 130 NRGs were shown in S1 Table. Comprehensive details regarding the sample collection and processing procedures, and associated metadata for the two GEO datasets employed (GSE134347 and GSE26440) are documented in Table 1.

**Table 1:** CEO microarray chip information.

	GSE134347	GSE26440
Platform	GPL17586	GPL570
Species	Homo sapiens	Homo sapiens
Tissue	Blood	Blood
Samples (Sepsis)	156	98
Samples (Control)	83	32
Reference	PMID: 33305733	PMID: 21738952
	PMID: 35903199	PMID: 26376786
		PMID: 24650276

GEO, Gene Expression Omnibus.

### Differentially expressed genes (DEGs) related to neutrophil inflammatory cell death in sepsis

According to the sample grouping of the Combined Dataset, the R package limma (Version 3.58.1) was used to perform differential analysis of genes in the Sepsis and the Control groups. The cut-off criteria were |logFC| > 1.0 and adj. *p* < 0.05. The *p* value correction method was the Benjamini-Hochberg procedure (BH). The results of the differential analysis were plotted using the R package ggplot2 (Version 3.4.4) for volcano plots. The DEGs were identified from the Combined Dataset that have |logFC| > 1.0 and adj. *p* < 0.05 through differential analysis. The intersection of these DEGs was taken with NRGs and a Venn diagram was created to obtain NRDEGs. The R package pheatmap (Version 1.0.12) was used to generate a heat map.

### Gene ontology (GO) and kyoto encyclopedia genes genomes (KEGG) pathway enrichment analysis

We performed GO and KEGG pathway enrichment analysis of NRDEGs using the R package clusterProfiler (Version 4.10.0). The criteria for adj. (*p* < 0.05) and FDR value (*q* value) <0.25 were set, and the *p* value correction method was the BH procedure.

### Gene set enrichment analysis (GSEA)

In this study, firstly, the genes of Combined Dataset were sorted according to the log FC value. Then, the R package clusterProfiler (Version 4.10.0) was used to perform GSEA on all genes in the Combined Dataset. The gene sets used for GSEA analysis were sourced from the Molecular Signatures Database (MSigDB), specifically the All Canonical Pathways collection (MSigDB v2022.1, file: c2.cp.all.v2022.1.Hs.symbols.gmt). During the GSEA analysis, a statistical method based on phenotype-label permutation was employed, with the permutation count set to 1,000 iterations. Parameters were as follows: the seed value set was 2024; no calculation count; with a minimum of 10 genes and a maximum of 500 genes in each gene set. Access to the c2 gene sets was obtained from MSigDB for GSEA; the selection criteria were adj. (*p* < 0.05) and FDR value (*q* value) < 0.25, and the *p* value correction method was the BH procedure.

### Protein-protein interaction (PPI) networks and the hub genetic screening

In this study, the application of the STRING database was based on NRDEGs, with the minimum correlation coefficient greater than 0.700 (high confidence) being used as the standard. Parameters were set to hide disconnected nodes in the network, and a PPI Network related to NRDEGs was constructed. In addition, five kinds of algorithms of Cytoscape software cytoHubba were applied: MCC (Maximal Clique Centrality), DMNC Centrality, DMNC (Density of Maximum Neighborhood Component), MNC (Maximum Neighborhood Component), and Degree and EPC (Edge Percolated Component). The NRDEGs’ scores were first calculated, then the top 10 NRDEGs were selected according to the score. At last, the intersection of the genes obtained from five different algorithms was taken and a Venn diagram was created for analysis. The intersected genes were considered hub genes related to NRGs

### Construction of control network

To analyze the relationship between neutrophilic inflammation cell death related-hub genes (hub genes) and miRNA, the TarBase database (http://www.microrna.gr/tarbase) was used to access the miRNA associated with neutrophilic inflammation cell death related-hub genes (hub genes), and the mRNA-miRNA regulatory network was visualized by cytoscape software.

In addition, transcription factor (TF) controls gene expression by interacting with neutrophilic inflammation cell related hub genes at the post-transcriptional stage. TFs retrieved from the ChIPBase database (https://rnasysu.com/chipbase3/index.php) were used to analyze their regulatory effects on the neutrophilic inflammation cell related hub genes (hub genes), and the mRNA-TF regulatory network was visualized using cytoscape software.

### Differential expression verification and ROC curve analysis of hub genes

A group comparison chart was drawn based on the expression of hub genes related to neutrophil inflammatory cell death from the combined dataset. Then, the R package pROC was used to plot the ROC curve of neutrophilic inflammation cell death-related hub genes, and the area under the curve was calculated to assess the diagnostic effectiveness of the expression levels of these hub genes for the occurrence of sepsis. The AUC of the ROC curve is generally between 0.5 and 1. An AUC greater than 0.5 indicates that the expression of the molecule has a trend to promote the occurrence of the event; the closer the AUC to 1, the better the diagnostic effect. An AUC between 0.5 and 0.7 indicates low accuracy, between 0.7 and 0.9 indicates moderate accuracy, and above 0.9 indicates high accuracy.

### Immune infiltration analysis of sepsis (CIBERSORT)

CIBERSORT is based on the principle of linear support vector regression to deconvolute the transcriptome expression matrix to estimate the composition and abundance of immune cells in a mixture of cells. Using the CIBERSORT algorithm in conjunction with the LM22 feature gene matrix, we filtered the output to retain data with immune cell enrichment scores greater than zero, ultimately obtaining the specific results of the immune cell infiltration matrix in the Combined Dataset. A bar chart was created to display the proportions. Subsequently, the R package ggplot2 (Version 3.4.4) was used to generate group comparison plots showing the expression differences of LM22 immune cells between the Sepsis and the Control groups in the Combined Dataset. Next, we selected immune cells that exhibited significant differences between the two groups for further analysis. Using the Spearman algorithm, we calculated the correlations between immune cells and employed the R package pheatmap (Version 1.0.12) to create a heat map that illustrates the correlation analysis results among the immune cells themselves. Additionally, we calculated the correlation between hub genes and immune cells based on the Spearman algorithm, retaining results with a *p*<0.05. The R package ggplot2 (Version 3.4.4) was then used to create a bubble chart to present the correlation analysis results between hub genes and immune cells.

### Statistical analysis

All data processing and analysis of this article were based on the R software (Version 4.3.0); unless otherwise specified, the statistical significance of continuous variables between two groups was estimated using the independent Student’s t-test for normally distributed variables, and the Mann-Whitney U test (Wilcoxon Rank Sum Test) was used to analyze differences between non-normally distributed variables. For comparisons involving three or more groups, the Kruskal-Wallis test was employed. Results were calculated using Spearman correlation analysis to determine the correlation coefficients between different molecules. For multiple comparison correction, the Benjamini-Hochberg (BH) method was implemented throughout the pipeline to adjust the false discovery rate (FDR), including differential expression analysis, GO/KEGG functional enrichment analysis, and GSEA. The default FDR threshold was set at 0.05. In certain functional pathway enrichment analyses, an FDR (*q* value) <0.25 was additionally applied as a complementary screening criterion. Unless specifically indicated, all statistical *p* values are two-tailed, with *p*<0.05 considered statistically significant.

### Ethics statement

The data used in this study were sourced from public, anonymized datasets in the NCBI GEO database. All data had identifiable personal information removed by the original research teams during the initial submission process. This complies with relevant institutional and international ethical guidelines. As this study constitutes secondary analysis of anonymized public database data and does not involve direct human subject information, additional ethics committee approval or informed consent is not required.

## Results

### Combined dataset and standardization

**Fig 1:**
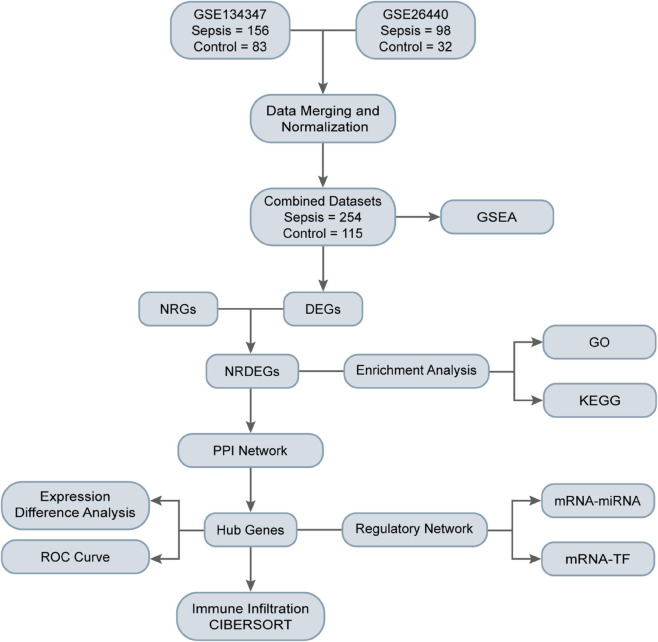
Flow chart for the comprehensive analysis of NRDEGs. GSEA, gene set enrichment analysis; DEGs, differentially expressed genes; NRGs, NETosis-related genes; NRDEGs, NETosis-related differentially expressed genes; GO, gene ontology; KEGG, Kyoto encyclopedia of genes and genomes; PPI, Protein-protein interaction; ROC curve, Receiver operating characteristic curve; TF, Transcription factor.

**Fig 2:**
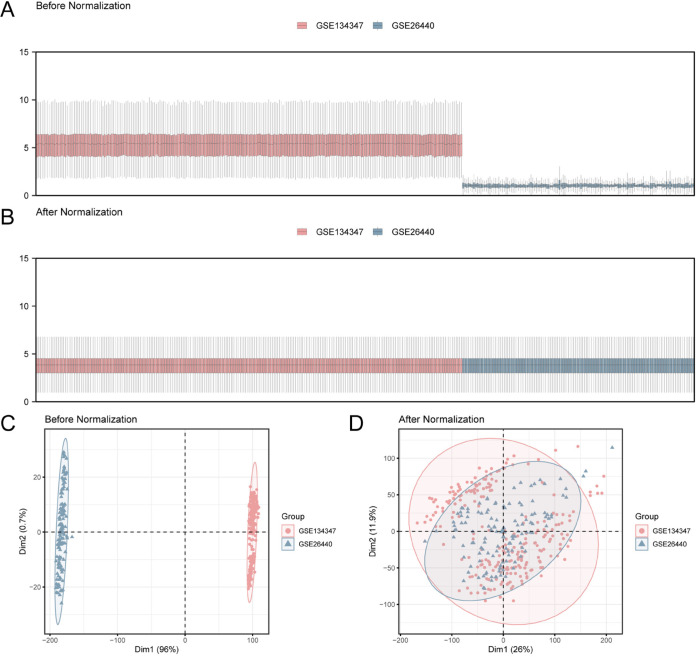
Batch effects removal of GSE134347 and GSE26440. A. before going to batch processing integrated GEO dataset (Combined Dataset) distribution box plot. B. Post-batch integrated GEO dataset (Combined Dataset) distribution boxplots. C. PCA plot of the datasets before debatching. D. to batch processing of the integration of GEO datasets (Combined Dataset) of PCA. PCA, Principal Component analysis; Red for sepsis GSE134347 dataset, blue for sepsis GSE26440 dataset.

The overall flow chart of this study is presented in Fig 1. First, the R package sva was used to remove batch effects from the Sepsis datasets GSE134347 and GSE26440, resulting in Combined Dataset. Subsequently, boxplots (Fig 2A and 2B) were used to compare the expression value differences of the datasets before and after batch effect removal. Additionally, PCA plots (Fig 2C and 2D) were used to compare the distribution of low-dimensional features of the datasets before and after batch effect removal. The results from the boxplots and PCA plots indicate that the batch effects in the sepsis datasets were largely eliminated after the batch effect removal process.

### Identification of NRDEGs

**Fig 3:**
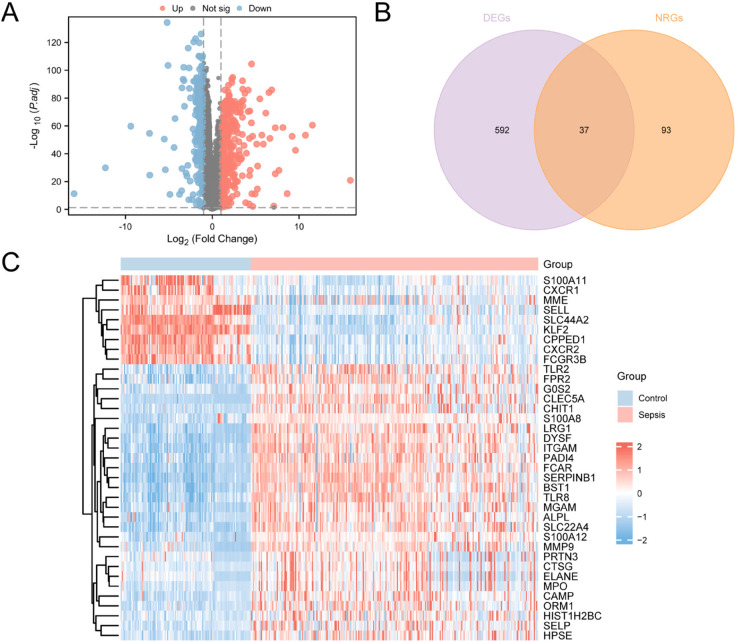
Differential gene expression analysis. A. Combined Dataset in Sepsis group and Control group volcanic diagram analysis of differentially expressed genes. B. Combined Dataset of DEGs and NRGs Venn diagram. C. Combined Dataset neutrophilic inflammation in NRDEGs heat maps. Pale red for Sepsis, light blue for Control. Red represents high expression in heat map, blue represents lower expression.

The Combined Dataset contain a total of 629 DEGs (S2 Table) that meet the criteria of |logFC| >1.0 and adj *p* < 0.05. Among these, there are 348 upregulated genes (|logFC| > 1.0 and adj *p* < 0.05 ) and 281 downregulated genes (logFC <–1.0 and adj *p*<0.05). A volcano plot was created based on the differential analysis results of this dataset (Fig 3A). The intersection of all DEGs with |logFC| > 1.0 and adj *p*<0.05 and NRGs was taken to create a Venn diagram (Fig 3B). This resulted in 37 NRDEGs, with specific information provided in S3 Table. Based on the intersection results, the expression differences of the NRDEGs across different sample groups in the Combined Dataset were analyzed, and a heat map was generated using the R package pheatmap to display the analysis results (Fig 3C).

### GO and KEGG pathway enrichment analysis

**Fig 4:**
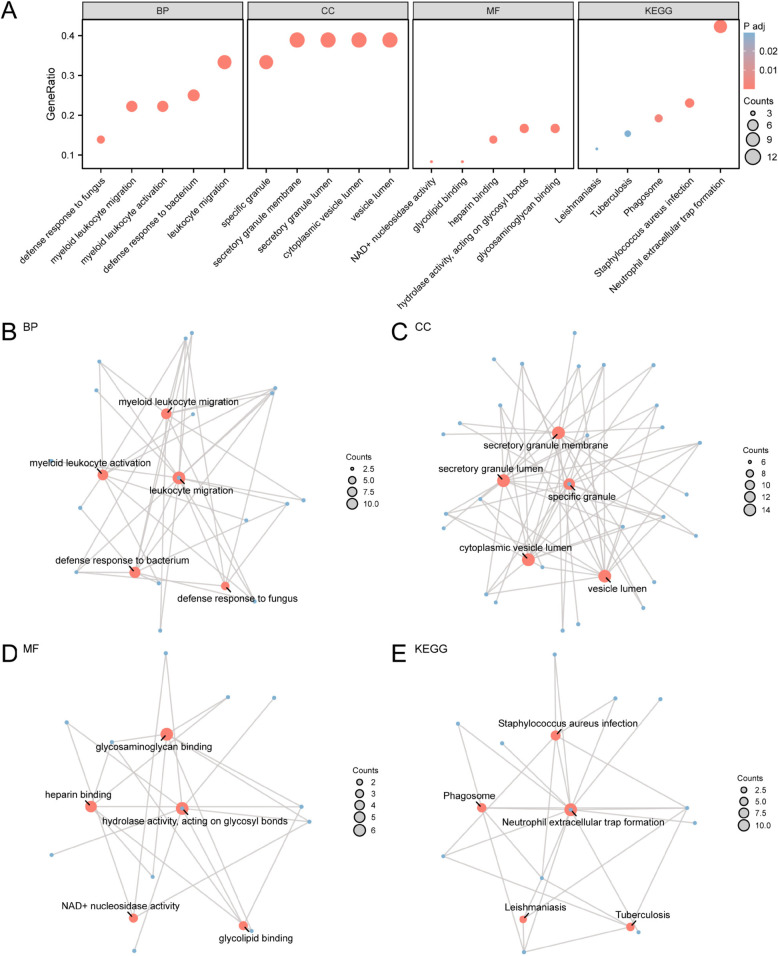
GO and KEGG enrichment analysis for NRDEGs. A. Bubble diagram of gene ontology (GO) and pathway (KEGG) enrichment analysis results of NRDEGs: biological process (BP), cell Component (CC), molecular function (MF) and biological pathway (KEGG). Abscissa to GO terms and KEGG terms. B-E. NRDEGs differentially expressed genes related to the gene ontology (GO) and pathway enrichment analysis results (KEGG) network diagram shows: BP (B), CC (C), MF (D) and KEGG (E). Red nodes represent items, blue nodes represent molecules, and the lines represent the relationship between items and molecules. GO KEGG selection criteria for adj. (*p* < 0.05) and FDR value (*q* value) < 0.25, *p* value correction methods for BH.

To further investigate the biological functions of NRDEGs, GO and KEGG functional enrichment analyses were performed, the results are shown in Table 2. The GO enrichment showed that the functions of the NRDEGs were mainly related to leukocyte migration, myeloid leukocyte migration, myeloid leukocyte activation, defense response to bacterium, defense response to fungus, and other biological processes (BP); cellular Components (CC) including specific granule, secretory granule membrane, secretory granule lumen, cytoplasmic vesicle lumen, and vesicle lumen; hydrolase activity, acting on glycosyl bonds, glycosaminoglycan binding, heparin binding, and NAD+ nucleosidase activity in molecular functions (MF). It was also enriched in KEGG pathways including glycolipid binding, NET formation, staphylococcus aureus infection, phagosome, leishmaniasis and tuberculosis. The results of the GO and KEGG pathway enrichment analysis were visualized using a bubble chart (Fig 4A). At the same time, according to the GO and KEGG pathway enrichment analysis, the network diagrams of BP, CC, MF, and biological pathway (KEGG) were drawn (Fig 4B–4E).

**Table 2:** Results of GO and KEGG enrichment.

Ontology	ID	Description	Gene Ratio	BgRatio	*p-value*	*p adjust*	*q-value*
BP	GO:0050900	leukocyte migration	1.236	384/18800	3.57747E-12	3.27696E-09	2.26699E-09
BP	GO:0097529	myeloid leukocyte migration	8.36	229/18800	9.67416E-09	3.27017E-06	2.26229E-06
BP	GO:0002274	myeloid leukocyte activation	8.36	232/18800	1.07102E-08	3.27017E-06	2.26229E-06
BP	GO:0042742	defense response to bacterium	9.36	364/18800	2.05626E-08	4.70884E-06	3.25755E-06
BP	GO:0050832	defense response to fungus	5.36	53/18800	5.17742E-08	9.48503E-06	6.56169E-06
CC	GO:0042581	specific granule	1.236	160/19594	6.11424E-17	4.29824E-15	2.4319E-15
CC	GO:0030667	secretory granule membrane	14.36	312/19594	1.39512E-16	4.29824E-15	2.4319E-15
CC	GO:0034774	secretory granule lumen	14.36	322/19594	2.16702E-16	4.29824E-15	2.4319E-15
CC	GO:0060205	cytoplasmic vesicle lumen	14.36	325/19594	2.4662E-16	4.29824E-15	2.4319E-15
CC	GO:0031983	vesicle lumen	14.36	327/19594	2.6864E-16	4.29824E-15	2.4319E-15
MF	GO:0016798	hydrolase activity, acting on glycosyl bonds	6.36	144/18410	3.31148E-07	4.04001E-05	2.05661E-05
MF	GO:0005539	glycosaminoglycan binding	6.36	234/18410	5.59867E-06	0.000341519	0.000173853
MF	GO:0008201	heparin binding	5.36	168/18410	1.78766E-05	0.000496433	0.000252714
MF	GO:0003953	NAD+ nucleosidase activity	3.36	281/18410	2.17522E-05	0.000496433	0.000252714
MF	GO:0051861	glycolipid binding	3/36	29/18410	2.42294E-05	0.000496433	0.000252714
KEGG	hsa04513	Neutrophil extracellular trap formation	11/26	190/8164	4.63165E-12	3.47374E-10	2.48647E-10
KEGG	hsa05150	Staphylococcus aureus infection	6/26	96/8164	4.30057E-07	1.61271E-05	1.15436E-05
KEGG	hsa04145	Phagosome	5/26	152/8164	0.000100456	0.002511393	0.001797629
KEGG	hsa05140	Leishmaniasis	3/26	77/8164	0.001794329	0.02960213	0.021188893
KEGG	hsa05152	Tuberculosis	4/26	180/8164	0.002337292	0.02960213	0.021188893

### GSEA of combined dataset genes

**Fig 5:**
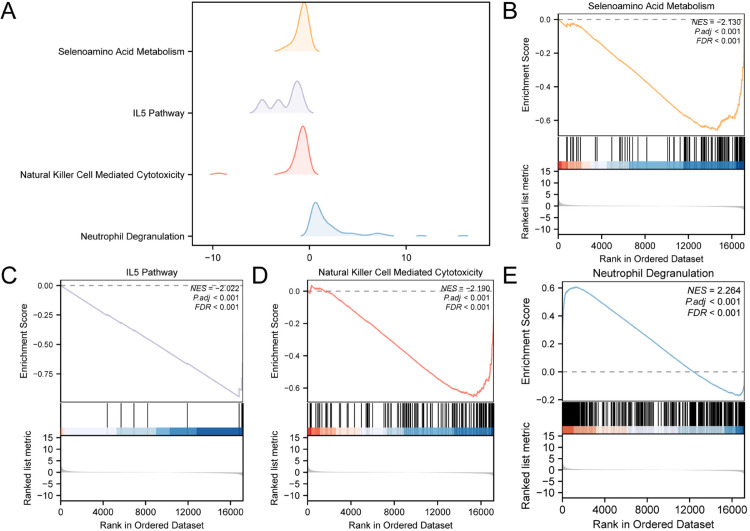
GSEA for combined dataset. A. four biological functions mountain map of GSEA of Combined Dataset. B-E. GSEA showed that all genes were significantly enriched in Selenoamino Acid Metabolism(B), IL5 Pathway(C), Natural Killer Cell Mediated Cytotoxicity(D). Neutrophil Degranulation (E). The screening criteria of GSEA were adj. *p* < 0.05 and FDR value (*q* value) < 0.25, and the *p* value correction method was BH.

To systematically investigate major functional and pathway alterations at the transcriptome-wide level in sepsis patients, we performed GSEA on all genes.The GSEA was conducted to investigate the relationships among the expression of all genes in the Combined Dataset, the BPs they participate in, the CC they affect, and the MF they perform (Fig 5A), with detailed results presented in Table 3. The results showed that all genes in the Combined Dataset were significantly enriched in Selenoamino Acid Metabolism (Fig 5B), IL5 Pathway (Fig 5C), Natural Killer Cell Mediated Cytotoxicity (Fig 5D), Neutrophil Degranulation (Fig 5E), and other biological functions and signaling pathways. These GSEA results not only delineate the panoramic landscape of immune dysregulation in sepsis, but also demonstrate strong concordance with subsequent GO/KEGG enrichment findings from NRDEGs. Both analyses converge on aberrant neutrophil functionality. Furthermore, the transcriptome-wide perspective afforded by GSEA corroborates the pivotal role of neutrophil dysfunction and its associated signal transduction pathways in the pathogenesis of sepsis. This integrated evidence provides a robust theoretical foundation and biological rationale for NRDEGs screening and functional interpretation.

**Table 3:** Results of GSEA for combined dataset.

Ontology	ID	Description	Gene Ratio	BgRatio	*p-value*	*p adjust*	*q-value*
BP	GO:0050900	leukocyte migration	1.236	384/18800	3.57747E-12	3.27696E-09	2.26699E-09
BP	GO:0097529	myeloid leukocyte migration	8.36	229/18800	9.67416E-09	3.27017E-06	2.26229E-06
BP	GO:0002274	myeloid leukocyte activation	8.36	232/18800	1.07102E-08	3.27017E-06	2.26229E-06
BP	GO:0042742	defense response to bacterium	9.36	364/18800	2.05626E-08	4.70884E-06	3.25755E-06
BP	GO:0050832	defense response to fungus	5.36	53/18800	5.17742E-08	9.48503E-06	6.56169E-06
CC	GO:0042581	specific granule	1.236	160/19594	6.11424E-17	4.29824E-15	2.4319E-15
CC	GO:0030667	secretory granule membrane	14.36	312/19594	1.39512E-16	4.29824E-15	2.4319E-15
CC	GO:0034774	secretory granule lumen	14.36	322/19594	2.16702E-16	4.29824E-15	2.4319E-15
CC	GO:0060205	cytoplasmic vesicle lumen	14.36	325/19594	2.4662E-16	4.29824E-15	2.4319E-15
CC	GO:0031983	vesicle lumen	14.36	327/19594	2.6864E-16	4.29824E-15	2.4319E-15
MF	GO:0016798	hydrolase activity, acting on glycosyl bonds	6.36	144/18410	3.31148E-07	4.04001E-05	2.05661E-05
MF	GO:0005539	glycosaminoglycan binding	6.36	234/18410	5.59867E-06	0.000341519	0.000173853
MF	GO:0008201	heparin binding	5.36	168/18410	1.78766E-05	0.000496433	0.000252714
MF	GO:0003953	NAD^ + ^ nucleosidase activity	3.36	281/18410	2.17522E-05	0.000496433	0.000252714
MF	GO:0051861	glycolipid binding	3/36	29/18410	2.42294E-05	0.000496433	0.000252714
KEGG	hsa04513	Neutrophil extracellular trap formation	11/26	190/8164	4.63165E-12	3.47374E-10	2.48647E-10
KEGG	hsa05150	Staphylococcus aureus infection	6/26	96/8164	4.30057E-07	1.61271E-05	1.15436E-05
KEGG	hsa04145	Phagosome	5/26	152/8164	0.000100456	0.002511393	0.001797629
KEGG	hsa05140	Leishmaniasis	3/26	77/8164	0.001794329	0.02960213	0.021188893
KEGG	hsa05152	Tuberculosis	4/26	180/8164	0.002337292	0.02960213	0.021188893

GSEA, gene set enrichment analysis.

### PPI network of NRDEGs and screening of hub genes

**Fig 6:**
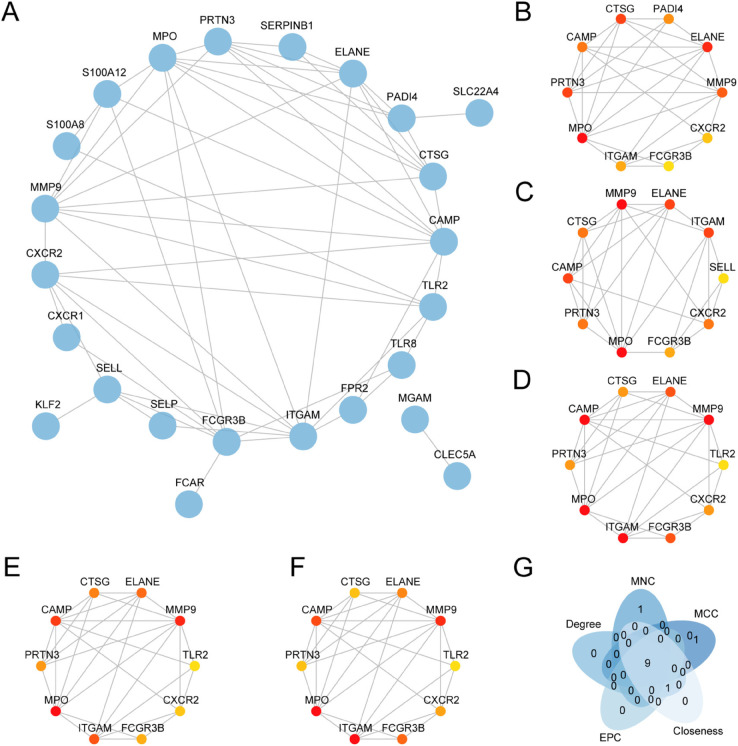
PPI network and hub genes analysis. A. STRING database calculation of neutrophils differentially expressed genes related to inflammation cell death protein-protein interaction network. B-F. CytoHubba plug-in calculated need neutrophils differentially expressed genes related to inflammation cell death of PPI network, including the MCC (B), newsun focus (C), Degree (D), EPC (E) and Closeness (F). G. CytoHubba need the five kinds of algorithms of the plugin neutrophils differentially expressed genes related to inflammation cell death (NRDEGs) Venn diagram. PPI network, interaction network; NRDEGs, differentially expressed genes.

The PPI network for a total of 37 NRDEGs (Fig 6A) was constructed using the STRING database. The PPI network showed that 25 NRDEGs had interaction relationships (S4 Table). Then, the top 10 NRDEGs from five algorithms were selected to construct PPI networks, respectively: MCC (Fig 6B), MNC (Fig 6C), Degree (Fig 6D), EPC (Fig 6E), and Closeness (Fig 6F). Finally, the intersection of the genes from five algorithms is shown in the Venn diagram (Fig 6G) for analysis, and the intersected genes were the hub genes for sepsis, comprising nine hub genes: *MPO*, *ELANE*, *PRTN*3, *CTSG*, *MMP*9, *CAMP*, *ITGAM*, *CXCR*2, and *FCGR*3*B*.

### Construction of mRNA-miRNA and mRNA-TF regulatory networks

**Fig 7:**
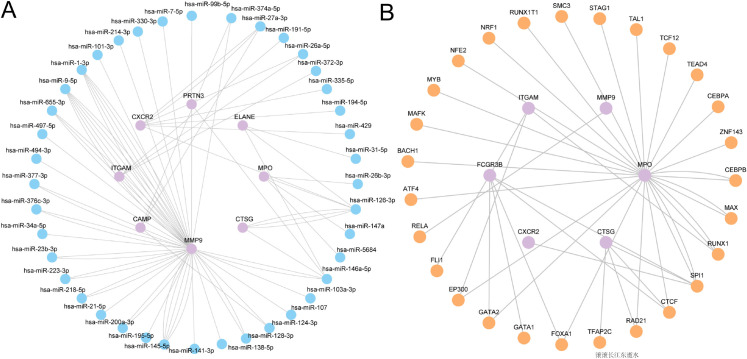
Regulatory network of NRDEGs. A. Genetic variations associated with neutrophilic inflammation cell death mRNA - miRNAs regulation Network. B. Neutrophils genetic variations associated with inflammatory cell death mRNA-TF control network (mRNA-TF regulatory network). mRNA are shown in purple, miRNAs in blue, and TFs in orange.

To further investigate the upstream regulatory mechanisms of NRDEGs in sepsis, we constructed mRNA-miRNA and mRNA-TF regulatory networks. The miRNAs associated with NRDEGs were obtained from TarBase database to build an mRNA-miRNA regulatory network (Fig 7A), which contains 8 NRDEGs and 76 miRNAs (S5 Table). This network revealed that core genes such as *MMP*9, *CXCR*2, and *ITGAM* were regulated by multiple miRNAs, suggesting miRNAs likely play critical roles in modulating the expression of these key genes. For instance, *MMP*9 demonstrated tight regulatory relationships with several miRNAs (e.g., hsa-miR-145-5p, hsa-miR-223-3p), potentially influencing its functions in inflammatory responses and cellular migration. Additionally, through screening the ChIPBase database, the TFs combined with NRDEGs were obtained from the ChIPBase database to build the mRNA-TF regulatory network (Fig 7B), which contains 6 NRDEGs and 41 TFs (S6 Table). This network uncovered regulatory interactions among genes like *MPO*, *CXCR*2, *ITGAM*, *SPI*1, *SPI*1 and *EP*300, suggesting these TFs may participate in sepsis-related immune responses and inflammatory regulation by modulating the transcriptional activity of core genes.

### Differential expression verification and ROC curve analysis of hub genes

**Fig 8:**
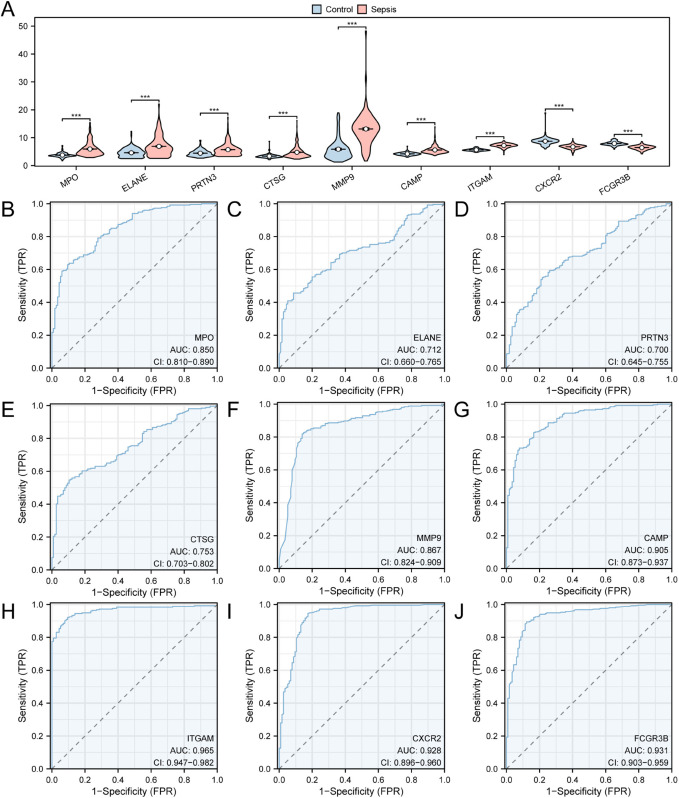
Expression difference and ROC curve analysis. A. The hub genes linked to the neutrophilic inflammation cell death (hub genes) in the integration of Combined Dataset the grouping comparison chart. B-J: Expression values have significant differences in group comparison chart of neutrophilic inflammation cell death related to the hub genes: MPO(B), ELANE(C), PRTN3(D), CTSG(E), MMP9(F), CAMP(G), ITGAM(H), CXCR2(I), FCGR3B and(J) of the ROC curve. ROC, Receiver Operating Characteristic Curve. On behalf of *p* value < 0.001, highly statistically significant. The closer AUC is to 1, the better the diagnosis results, AUC in 0.5 ∼ 0.7 with low accuracy, and AUC in 0.7 ∼ 0.9 has a certain accuracy. Grouping is red for Sepsis group, blue for Control group.

We further explored the expression levels of the nine hub genes in the Sepsis and Control groups in Combined Dataset, which had significant statistical significance (*p* < 0.001 ) (Fig 8A). Next, the ROC curves (Fig 8B–8J) show that the *ITGAM*, *CXCR*2, and *FCGR*3*B* expression levels provide high accuracy for classifying the Sepsis and Control group (AUC > 0.9); *MMP*9, *MPO*, and *CTSG* expression levels exhibit moderate accuracy for this classification (0.7 < AUC < 0.9).

### Correlation analysis between hub genes and core immune cells in sepsis

**Fig 9:**
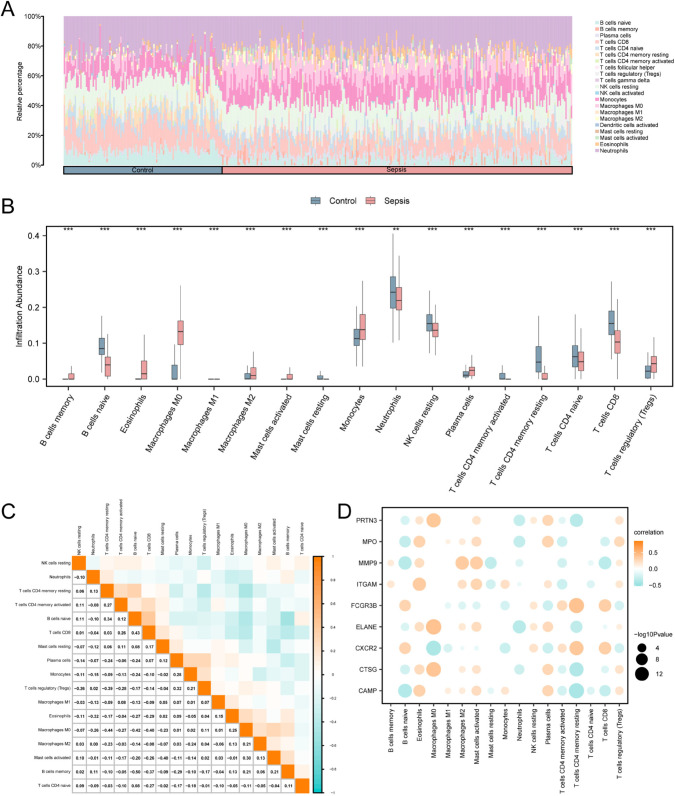
Combined dataset immune infiltration analysis by CIBERSORT algorithm. A - B. Immune cells in the integration of Combined Dataset of than histogram (A) and (B) grouping comparison chart. C. Correlation heatmap of immune cell infiltration abundance in the integrated Combined Dataset. D. Hub genes in the integration of Combined Dataset and immune cell infiltration in abundance the relevance of the bubble chart. ** on behalf of the p < 0.01, there is highly statistically significant; *** represents p < 0.001 and highly statistically significant. The absolute value of correlation coefficient (r value) below 0.3 was weak or no correlation, between 0.3 and 0.5 was weak correlation, between 0.5 and 0.8 was moderate correlation, and above 0.8 was strong correlation. Red for Sepsis group, blue for Control group. Is positively related to green as the negative correlation, orange, color shades on behalf of the correlation.

Using the Combined Dataset, the CIBERSORT algorithm was applied to calculate the infiltration abundance of 21 types of immune cells within the dataset (Fig 9A). Further, the expression levels of 17 immune cells were statistically significant between the Sepsis and the Control groups (*p* < 0.05) (Fig 9B), respectively: B cells memory, B cells naive, Eosinophils, Macrophages M0, Macrophages M1, Macrophages M2, Mast cells activated, Mast cells resting, Monocytes, Neutrophils, NK cells resting, Plasma cells, T cells CD4 memory activated, T cells CD4 memory resting, T cells CD4 naive, T cells CD8, and T cells regulatory (Tregs).

We used immune infiltration analysis to determine the correlation of expression abundance of 17 immune cells in the Combined Dataset (Fig 9C). The results showed that B cells naive and T cells CD8 exhibited the strongest positive correlation (r value = 0.43), while B cells memory and B cells naive demonstrated the strongest negative correlation (r value = -0.50). Finally, a correlation bubble chart illustrated the relationships between hub genes and immune cell infiltration abundance in the Combined Dataset (Fig 9D). *MMP*9 showed a significant positive correlation with Macrophages M0 (r value > 0.85, *p* < 0.05); *FCGR*3*B* displayed a significant negative correlation with Macrophages M0 (r value < –0.53, *p* < 0.05).

## Discussion

We screened 629 DEGs and 37 NREDGs and identified biological functions and significant pathways associated with sepsis. Through PPI analysis, nine potential key genes associated with neutrophils in sepsis (hub genes), namely *MPO*, *ELANE*, *PRTN*3, *CTSG*, *MMP*9, *CAMP*, *ITGAM*, *CXCR*2, and *FCGR*3*B*, were identified. Immune cell infiltration analysis illustrated the relationship between core immunes and sepsis.

During sepsis, the hub of the "cytokine storm" releases high levels of pro-inflammatory cytokines and proteases in the initial phase of sepsis [12]. Previous studies have emphasized the crucial role of neutrophils in the immune response, particularly their ability to generate NET to capture and neutralize pathogens [8]. NET are made of decondensed chromatin that forms weblike DNA structures, and they are coated with nuclear proteins including histones, granule proteins, cytosolic proteins, neutrophils, eosinophils,etc. [13] NETosis is a specialized form of neutrophil-mediated programmed cell death (distinct from apoptosis or necrosis) characterized by the expulsion of neutrophil extracellular traps(NET). NETosis plays a critical protective role in antimicrobial defense; its excessive or dysregulated activation can lead to uncontrolled inflammatory responses, exacerbating tissue damage, organ dysfunction, and sepsis progression. Thus, NETosis exhibits a "double-edged sword" effect in sepsis: it eliminates pathogens while potentially aggravating host inflammatory injury [14,15]. In our research, we identified a total of 37 genes that were specifically associated with neutrophil-related cell death. We observed aberrant expression patterns in sepsis patients, suggesting that imbalanced NETosis regulation may underlie immune dysregulation in sepsis. The differential expression of NRDEGs might indicate altered neutrophil responses, potentially enhancing antimicrobial capacity while concurrently increasing the risk of inflammation-associated tissue damage, thus providing insight into early diagnosis and potential therapeutic targets for modulating immune responses in sepsis.

To determine the fundamental biological functions of these NRDEGs, we performed functional enrichment analysis (GO and KEGG). The results revealed that the NRDEGs were predominantly associated with pathways related to NET formation, cellular phagocytosis, and lymphocyte migration. This highlights the active engagement of neutrophils in these critical immune processes during sepsis. The association of NRDEGs with NETosis suggests that neutrophils might contribute both beneficially and detrimentally to the inflammatory response in sepsis [16]. Investigating the balance between these opposing effects through targeted modulation of NRDEGs might provide new avenues for therapeutic intervention, particularly in sepsis where the immune response is often dysregulated.

As a supplementary method, we performed GSEA. The results showed that all genes (Combined Dataset) play key roles in Selenoamino Acid Metabolism, IL5 Pathway, Natural Killer Cell Mediated Cytotoxicity, and Neutrophil Degranulation. Selenoamino acid metabolism is associated with the regulation of neutrophil function, but its precise role in sepsis remains to be further elucidated. Studies have indicated that selenium supplementation could enhance neutrophil function, including degranulation, by promoting the production of selenoproteins that are involved in antioxidant defense mechanisms [17]. While the IL-5 signaling pathway primarily participates in eosinophil activation, some studies suggest that it may also influence neutrophil activity and migration under specific inflammatory conditions [18]. However, direct evidence linking the IL-5 signaling pathway to sepsis is currently limited. The enrichment of this pathway in our study may more reflect the complexity of the inflammatory response network, and its biological significance requires further experimental validation. In neutrophils, IL-5 signaling has been shown to influence their activation and migration, although the exact mechanisms remain less clear compared to eosinophils. Studies have indicated that IL-5 might augment neutrophil responses during allergic inflammation, potentially exacerbating tissue damage and contributing to the pathophysiology of conditions such as asthma [19]. Furthermore, the interplay between IL-5 and other cytokines in the inflammatory milieu could shape the functional outcomes of these immune cells, highlighting the complexity of IL-5’s role in immune regulation. Recent studies have highlighted the importance of neutrophil degranulation in various pathological conditions, including myocardial infarction and infectious diseases, underscoring its relevance in clinical settings [20]. In this study, GSEA, employing a genome-wide approach, systematically identified major sepsis-associated pathway alterations and provided a theoretical basis for subsequent investigations into NRDEGs and immune mechanisms. The results demonstrated strong concordance between GSEA and core gene enrichment analyses, forming a complementary and mutually reinforcing relationship that further substantiates the pivotal roles of core genes in sepsis immune regulation. By emphasizing GSEA’s global corroborative and supportive function, this integrated approach reinforces the research rationale and clarifies the central investigative trajectory.

We further investigated the interaction among NRDEGs, and we selected nine hub genes. This study employed a comprehensive approach to hub gene selection by integrating multiple PPI network centrality metrics, each emphasizing distinct dimensions of node importance in the network [21]. For instance, Degree centrality reflects the number of direct interactions between a gene and other proteins, MNC and MCC identify genes’ roles in densely connected subnetworks, EPC evaluates edge penetration between proteins, and Closeness measures a gene’s efficiency in information propagation within the network. The nine hub genes consistently performed well across these metrics, enhancing the robustness and scientific validity of the selection. This multi-dimensional methodology facilitates the identification of core molecules critical to both network structure and biological function, laying a foundation for subsequent mechanistic and translational research.

*ITGAM*, is a critical adhesion molecule involved in the immune response, particularly in leukocyte adhesion and migration to sites of inflammation. *ITGAM* interacts with various ligands, including iC3b and fibrinogen, facilitating the attachment of leukocytes to the vascular endothelium and their subsequent infiltration into tissues [22]. Dysregulation of *ITGAM* function could lead to impaired immune responses and contribute to the pathogenesis of autoimmune diseases and chronic inflammatory conditions. Understanding the precise mechanisms by which *ITGAM* mediates leukocyte adhesion and migration could provide insights into potential therapeutic targets for modulating immune responses in sepsis.

*CXCR*2 is a G protein-coupled receptor that plays a pivotal role in mediating the migration of leukocytes toward sites of inflammation by responding to specific chemokines, particularly those in the *CXC* family. Activation of *CXCR*2 triggers various intracellular signaling pathways that promote cell migration and activation, essential for effective immune responses [23]. Research has shown that *CXCR*2 signaling is critical in cancer biology, influencing tumor growth and metastasis by modulating the tumor microenvironment [24]. *CXCR*2 has been implicated in remyelination processes in preclinical models of demyelination, suggesting its potential as a therapeutic target in neurodegenerative diseases [25]. The intricate balance of *CXCR*2 signaling is vital for maintaining immune homeostasis, and dysregulation could lead to chronic inflammatory conditions and cancer progression [26].

*FCGR*3*B*, is an important receptor involved in the immune response, particularly in mediating antibody-dependent cellular cytotoxicity (ADCC). Variations in *FCGR*3*B* expression and function could significantly impact individual susceptibility to infections and the efficacy of vaccines. Recent studies have identified genetic polymorphisms within the *FCGR*3*B* gene that might modulate immune responses, including responses to HIV vaccines [27]. Additionally, *FCGR*3*B* is involved in the clearance of immune complexes and apoptotic cells, playing a role in maintaining immune homeostasis [28]. *CAMP*, an antimicrobial peptide, is upregulated in both infectious and non-infectious inflammatory states, and its expression is not exclusive to sepsis-similar changes, which can also be observed in other inflammation-related diseases (e.g., pneumonia, rheumatic diseases). Thus, the upregulation of *CAMP* in sepsis likely reflects general inflammatory activation rather than sepsis-specificity. Similarly, *FCGR*3*B* participates in immune responses to infections, with studies indicating altered expression in various infectious and autoimmune diseases, suggesting limited specificity as a diagnostic marker [29]. Although *CAMP* and *FCGR*3*B* were identified as hub genes in our PPI network and ranked highly across multiple centrality algorithms (including Degree, MCC, MNC, EPC, and Closeness), their diagnostic value as sepsis-specific biomarkers requires further validation in larger, multicenter clinical studies to avoid overinterpreting their specificity.

*MMP*9, plays a crucial role in extracellular matrix (ECM) remodeling, which is essential for various physiological and pathological processes, including wound healing, inflammation, and cancer metastasis. *MMP*9 is known for its ability to degrade type IV and type V collagen, which are integral Components of the basement membrane and interstitial matrix. Elevated levels of *MMP*9 have been associated with several malignancies, indicating its potential as a prognostic marker. Studies have shown that *MMP*9 is upregulated in breast cancer, where it correlates with poor patient survival outcomes, suggesting that its expression might facilitate tumor invasion and metastasis by remodeling the ECM to create pathways for cancer cell dissemination [30]. *MMP*9 is implicated in chronic inflammatory conditions, such as chronic obstructive pulmonary disease (COPD), where its activity could lead to structural changes in lung tissue [31]. The regulation of *MMP*9 expression and activity is thus a critical area of research, with potential therapeutic implications in targeting *MMP*9 to inhibit tumor progression and manage inflammatory diseases [32].

*MPO* is a heme-containing enzyme predominantly expressed in neutrophils and is crucial for the innate immune response. Recent studies have highlighted the dual role of *MPO*, acting as both a defender against infections and a potential mediator of tissue injury, which underscores its significance in the pathophysiology of inflammatory conditions [33]. Moreover, the regulatory networks involving [34] miRNAs and TFs associated with these hub genes further emphasize the complexity of the regulatory mechanisms at play.

The validation of hub genes identified in our study is critical for establishing their potential as biomarkers for sepsis. The diagnostic value of the core genes identified in this study was primarily based on the combined analysis of two public datasets (GSE134347 and GSE26440). The ROC curve analysis revealed that *ITGAM*, *CXCR*2, and *FCGR*3*B* exhibited high classification accuracy (AUC >0.9) between the Sepsis and Control groups(Fig 8), indicating their strong potential for early diagnosis. However, it should be noted that these AUC values are specific to the GSE134347 and GSE26440 datasets used in this study, and their generalizability to independent cohorts requires further validation. The significance of these findings aligns with previous studies that have highlighted the role of these genes in immune response and inflammation. For instance, *ITGAM* is crucial for leukocyte adhesion and migration, and its upregulation has been associated with enhanced inflammatory responses in sepsis [35]. Similarly, *CXCR*2, a chemokine receptor, plays a pivotal role in neutrophil recruitment to sites of infection, and its dysregulation has been implicated in the pathophysiology of sepsis [34,36]. *FCGR*3*B*, which encodes a receptor for the Fc region of immunoglobulin G, is involved in ADCC and has been shown to influence the severity of sepsis. The high sensitivity and specificity of these hub genes suggest that they could be integrated into clinical diagnostic protocols, potentially improving the accuracy and efficiency of sepsis identification. Notably, the hypothesis proposing *ITGAM* and *CXCR*2 as therapeutic targets demands cautious consideration [37]. While targeted inhibition has demonstrated amelioration of inflammatory injury in animal models, it may concurrently carry risks such as compromised pathogen clearance. Existing studies indicate that *CXCR*2 blockade can mitigate experimental sepsis-associated inflammation and organ damage [9], yet it may also impair the host’s immune defense against infection [38]. Consequently, subsequent functional experiments and clinical studies are urgently warranted to further validate the feasibility and safety of *ITGAM* and *CXCR*2-targeted interventions for sepsis treatment. *FCGR*3*B* not only showed upregulated expression in sepsis but was also elevated in other inflammatory diseases, indicating potential nonspecificity as a diagnostic biomarker. Thus, we recommend combining *FCGR*3*B* with other key indicators to improve diagnostic specificity and accuracy. Furthermore, the exploration of these biomarkers in diverse populations could enhance their applicability and effectiveness across different clinical settings. Future research should focus on elucidating the mechanisms by which these genes contribute to sepsis pathogenesis and their potential roles in guiding therapeutic interventions. Overall, the validation of these biomarkers represents a significant step towards improving early detection and management of sepsis, ultimately aiming to reduce morbidity and mortality associated with this critical condition.

This study presents several limitations that warrant consideration. Notably, the lack of functional validation at the laboratory level restricts the rigor of the current conclusions. Although potential key genes and biomarkers associated with NETosis were identified through multi-group bioinformatic analysis, these findings urgently require experimental confirmation to substantiate their validity. To address this limitation, future research could employ various experimental techniques to validate the results. For instance, real-time quantitative PCR (qPCR) could be utilized to detect mRNA expression levels of key genes, thereby confirming their transcriptional changes in sepsis samples. Enzyme-linked immunosorbent assay (ELISA) could be employed to assess protein-level alterations, thereby verifying the functional roles of differentially expressed genes at the protein level. Furthermore, flow cytometry could be applied to quantitatively analyze the functional status of neutrophil NETosis. The relatively small sample size might also restrict the generalizability of the identified gene expression patterns and their clinical relevance. Furthermore, potential batch effects inherent in the diverse datasets utilized could introduce variability, complicating the interpretation of the results. The lack of longitudinal clinical validation further constrains the applicability of the identified biomarkers in different patient populations. This study primarily employed differential expression analysis and PPI network analysis to systematically investigate molecular mechanisms. Weighted gene co-expression network analysis (WGCNA) is a powerful tool for identifying gene modules associated with disease phenotypes, enabling precise correlation between molecular subgroups and clinical characteristics. Given that this study has already incorporated multi-level functional and phenotypic analyses, WGCNA was not included in the current framework. However, we plan to integrate WGCNA in future studies to systematically explore functional modules and regulatory networks related to sepsis and NETosis. This approach will further support mechanistic insights and targeted therapeutic interventions [39]. Future studies should integrate larger-scale cohorts and multi-center clinical samples, supplemented with systematic experimental validation, to ensure the clinical applicability and generalizability of the identified biomarkers.

## Conclusion

This research elucidates the molecular mechanisms underlying NETosis in sepsis and identifies several potential biomarkers with promising implications for early diagnosis and treatment. The integration of bioinformatics analyses has facilitated the discovery of key genes and pathways associated with neutrophil inflammation and cell death. These findings not only enhance our understanding of sepsis pathophysiology but also pave the way for the development of targeted therapeutic strategies. Future investigations should focus on validating these biomarkers in clinical settings to establish their efficacy and reliability, ultimately contributing to improved patient outcomes in sepsis management.

## Supporting information

S1 Table NRGs. (XLSX)

S2 Table DEGs. (XLSX)

S3 Table NRDEGs. (XLSX)

S4 Table NRDEGs PPI. (XLSX)

S5 Table mRNA-miRNA. (XLSX)

S6 Table mRNA-TF. (XLSX)
